# Polyphenylene Sulfide Ultrafine Viscous Fibrous Membrane Modified by ZIF-8 for Highly Effective Oil/Water Separation under High Salt or Alkaline Conditions

**DOI:** 10.3390/membranes12101017

**Published:** 2022-10-20

**Authors:** Wenlei Liu, Lingli Yu, Xianfeng Cui, Ce Tan, Mengen Zhang, Di Wu, Zhenhuan Li, Maliang Zhang

**Affiliations:** 1 State Key Laboratory of Separation Membranes and Membrane Processes, National Center for International Joint Research on Separation Membranes, Tiangong University, Tianjin 300387, China; 2 School of Material Science and Engineering, Tiangong University, Tianjin 300387, China; 3 Tianjin Taipu Pharmaceutical Co., Ltd., Tianjin 300462, China; 4 State Key Laboratory of Drug Delivery Technology and Pharmacokinetics, Tianjin Institute of Pharmaceutical Research, Tianjin 300301, China; 5 Shandong Provincial Key Laboratory of Olefin Catalysis and Polymerization, Binzhou 256500, China

**Keywords:** polyphenylene sulfide, ZIF-8, oil/water separation, membrane, chemical resistance

## Abstract

The oil/water separation in harsh environments has always been a challenging topic all over the world. In this study, the ZIF-8/PPS fiber membranes were fabricated via the combination of hot pressing and in situ growth. The distribution of ZIF-8 in the membranes was adjusted by changing the ZIF-8 in situ growth time, which could control the oil/water separation effect. Due to the hydrophilic nature of the ZIF-8/PPS fiber membranes, the water molecules in the oil-in-water emulsion could quickly penetrate into the fiber membrane under the drive of pressure, gravity, and capillary force, forming a water layer on the surface of the fiber membranes. The coupling of the water layer and the fiber structure prevented direct contact between the oil molecules and the fiber membrane, thereby realizing the separation of the emulsion. The results show that when the ZIF-8 in situ growth time was 10 h, the contact angle, the porosity, and the pure water flux of the ZIF-8/PPS fiber membranes were 72.5°, 52.3%, and 12,351 L/h·m^2^, respectively. More importantly, the separation efficiency of M10 was 97%, and the oil/water separation efficiency reached 95% after 14 cycles. This study provides a novel strategy for preparing MOFs/fiber materials for oil/water separation in harsh environments.

## 1. Introduction

In recent years, due to the increase in industrial wastewater and the frequent occurrence of oil spills at sea, oily wastewater has caused serious water pollution and social problems [1,2,3,4]. Therefore, the treatment of oily wastewater in daily life and industrial production has become an urgent global problem [5,6]. Oil/water separation has also become a hot topic of research [7,8,9,10]. At present, the commonly used oil/water separation methods include adsorption, skimming, filtration, microbial degradation, and combustion [11,12]. However, these methods have problems such as high cost, poor separation efficiency, and ease to cause secondary pollution. Modern scientific researchers have developed a variety of new separation methods on the basis of existing separation methods. Among them, membrane separation technology has entered the field of view of scientists due to its low energy consumption, strong selectivity, good adaptability, and high filtration accuracy. Membrane separation and membrane processes have been widely used in the field of sewage resource utilization, and have become an indispensable part in the field of water resource utilization and environmental protection. Traditional membrane materials have poor high-temperature resistance or corrosion resistance, and have problems such as poorer dimensional stability, lower separation performance, and shorter service life in harsh environments. Currently, key issues such as the recovery of organic solvents, the treatment of highly corrosive sewage, the filtration of high-temperature flue gas, and the separation of small-sized industrial wastes have become hotspots of concern in many countries. Therefore, the development of special separation membrane materials that can withstand high temperatures, corrosion, and oxidation in harsh environments is an important means for future environmental protection, energy saving, emission reduction, and resource recovery.

**Figure 1:**
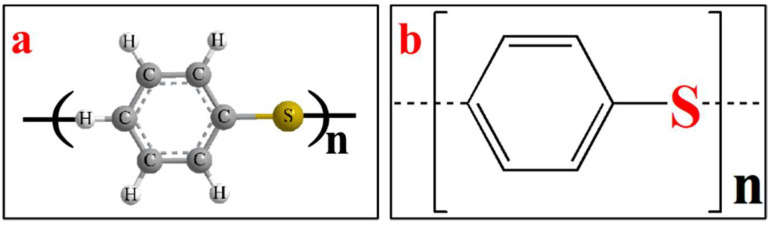
(**a**) Ball and stick model of PPS structure; (**b**) the chemical structure of PPS.

As a new material, polyphenylene sulfide (PPS) is widely used in the fields of purification, catalysis, advanced manufacturing, and electrochemistry [13,14,15,16]. Because PPS has excellent properties such as high-temperature resistance, corrosion resistance, balanced mechanical properties, and certain catalytic properties, it has been widely used successfully in many fields [17,18,19,20]. Since the start of the 21st century, researchers have conducted endless research on PPS [21,22,23,24]. With the continuous deepening of research on PPS by scientific researchers, PPS has broad prospects for development in the future. PPS is a semi-crystalline polymer. As shown in Figure 1, PPS has a thiol group in its molecular backbone. Due to the special structure of PPS and the excellent performance of high-temperature resistance and corrosion resistance, PPS has been made into porous materials for high-temperature dust removal, battery separators, and water treatment [25,26,27], such as PPS membrane, PPS non-woven fabric, and so on. In recent years, research on PPS membranes has become popular. Gao [28,29] composited graphene oxide (GO) and PPS to prepare a series of high-performance PPS-based membrane materials. Wang [30] chose diphenyl ether (DPE) and benzophenone (DPK) as diluents, and prepared PPS microporous membranes via TIPS, and the influence of the ratio of two diluents in the casting solution on the micropore structure of membranes was studied. The results showed that the structure of the membrane prepared by mixed diluent was quite different from that of pure diluent. In addition to PPS flat membranes, PPS fiber membranes are also widely prepared and applied. PPS fiber material is a high-performance material obtained by melt-blown technology, which has excellent heat resistance, corrosion resistance, and dimensional stability. Therefore, PPS fibers can be prepared into PPS fiber membranes through post-treatment processes (for example, hot pressing, etc.). PPS fiber membranes have high porosity, large specific surface area, and controllable structure. They are widely used in industry, agriculture, medicine, and other fields. More importantly, because PPS fiber membranes have a complex irregular multilayer network structure, the PPS fiber membranes have sufficient specific surface area to provide sites for the growth of nanoparticles; on the other hand, PPS can contact the droplets from different angles [31,32]. The characteristics make it have excellent hydrophilicity and liquid retention rate. Wang [33] also used vacuum filtration technology to prepare a composite membrane composed of a multi-walled carbon nanotube (MWCNTs) layer and a polyphenylene sulfide/cellulose (PPS/FC) fiber membrane. The MWCNTs layer is in the 400–1200 nm wavelength range. It has a high solar absorption rate (about 93%) and good light-to-heat conversion ability. The porous network structure PPS/FC membrane at the bottom had excellent water transmission ability, high-temperature stability, and good heat insulation performance. In addition, researchers have also developed a great interest in filter bags made of PPS fibers [34,35]. In summary, as a new type of membrane material, various PPS fiber membranes have attracted more and more attention.

Metal organic frameworks (MOFs) are a novel class of porous and crystalline materials fabricated by individual or multiple metal ions with organic linkages. As a new type of porous material with excellent properties, they are widely used in many fields [36,37,38]. The ZIF-8 is widely used in molecular sieving, sensors, gas storage, drug release, and heterogeneous catalysis due to its large specific surface area, adjustable pore size, and good chemical stability [39]. However, since ZIF-8 exists in crystalline form, it cannot be easily recycled. In order to improve the recyclability and applicability of ZIF-8 in use conditions, the combination of a suitable matrix and ZIF-8 is an effective strategy [40,41]. PPS fiber membranes have a unique fluffy laminated network structure, and their large specific surface area can provide enough growth sites for ZIF-8, and then realize the efficient and uniform dispersion of ZIF-8 in PPS fiber membranes.

Some studies show that fiber membranes can play a huge role in oil/water separation [42,43]. In this study, the melt-blown technology was used to prepare the virgin PPS non-woven fabric, and then the fluffy PPS non-woven fabric was subjected to hot pressing to obtain the PPS fiber membranes. In order to realize the good growth of ZIF-8 nanoparticles on the PPS fiber membranes, we nitrified the PPS fiber membranes [44], and the influence of ZIF-8 on the structure and performance of the fiber membranes were studied. The development of modern industry made certain oil/water separation environments under extreme conditions such as strong acid and alkali; based on this condition, we verified whether the prepared super-hydrophilic ZIF-8/PPS fiber membranes could maintain the original super-hydrophilic performance in harsh environments, which showed strong separation performance in oil/water separation. This study provides a novel strategy for preparing MOFs/fiber materials for oil/water separation in harsh environments. The novelty of our work is as follows:

- ZIF-8/PPS fiber membranes displayed the controllable nano/micro-scale pore structure.
- ZIF-8/PPS fiber membranes exhibited excellent water permeability and oil rejection.
- ZIF-8/PPS fiber membranes showed high-temperature resistance.
- ZIF-8/PPS fiber membranes exhibited excellent cycling performance.
- ZIF-8/PPS fiber membranes possessed excellent chemical durability under harsh conditions.

## 2. Experimental Procedure

### 2.1. Materials

PPS resin was obtained from Ticona (0320). Nitric acid and zinc nitrate were purchased from China National Pharmaceutical Group Corporation (Beijing, China). 2-Methylimidazole, silicone oil, phenyl silane, and hexadecane were obtained from Shanghai Macklin Biochemical Co., Ltd. (Shanghai, China). Chlorobenzene, methanol, and ethanol absolute were purchased from Tianjin Fengchuan Chemical Reagent Technology Company (Tianjin, China). Various oil-in-water emulsions and ultrapure water were made in our lab.

### 2.2. Preparation of PPS Non-Woven Fabrics

PPS non-woven fabrics were prepared via the melt-blowing method. Firstly, the PPS raw material was dried in a vacuum oven at 120 °C for 24 h. Secondly, the vacuum-dried PPS resin was melted and plasticized into a fluid melt through the heating zone of the screw extruder. The PPS melt flowed into the conical spinneret after passing through the metering pump, and PPS was pulled by the high-speed hot air on both sides of the spinneret to form a web. Finally, the ultrafine fibers in the web were thermally bonded to form PPS non-woven fabrics.

### 2.3. Nitrification Reaction and Hot-Pressing Process

The nitric acid solution was made up of 40 mL concentrated nitric acid and 40 mL water. The dried non-woven fabrics were immersed in the nitric acid solution at 60 °C for 2 h. Then, the non-woven fabrics were washed to neutral with deionized water. Finally, the nitrated PPS non-woven fabric was dried in a vacuum oven at 60 °C and stored for characterization and evaluation.

The nitrated PPS non-woven fabrics were cut into square pieces of 10 cm × 10 cm. The PPS fiber membranes were obtained by the hot-pressing process under different hot-pressing temperatures and pressures. The hot-pressing pressure range was 5–10 MPa and the hot-pressing temperature range was 80–95 °C [45,46].

### 2.4. Preparation of ZIF-8/PPS Fiber Membranes

**Scheme 1:**
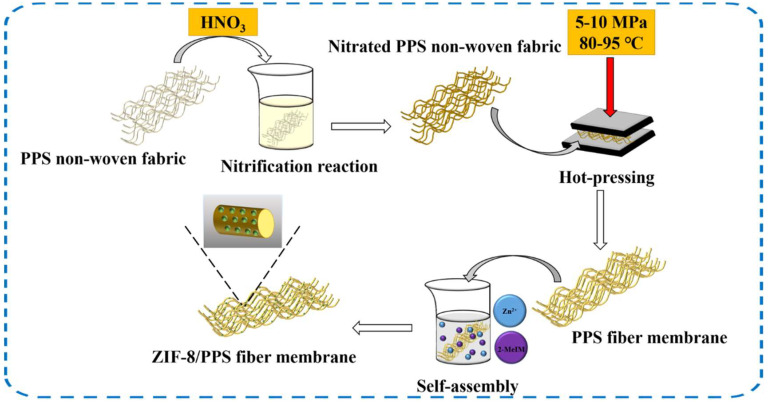
Schematically illustrated fabrication of ZIF-8/PPS fiber membranes.

An amount of 1.64 g of 2-Methylimidazole and 0.75 g of zinc nitrate were dissolved in 25 mL of methanol solution, respectively. Subsequently, the Zn^2+^ solution was added into the 2-Methylimidazole solution and stirred for 5 min to obtain a homogeneous solution. The PPS fiber membranes were immersed in the prepared homogeneous solution and allowed to stand for 4 h, 6 h, 8 h, 10 h, and 12 h, respectively. PPS fiber membranes allowed to stand for 4 h, 6 h, 8 h, 10 h, and 12 h were denoted as M4, M6, M8, M10, and M12, respectively. Finally, the ZIF-8/PPS fiber membranes were filtered twice by water and ethanol and then dried under vacuum at 60 °C for 12 h. The fabrication processes of ZIF-8/PPS fiber membranes are shown in Scheme 1.

### 2.5. Oil/Water Emulsion Separation Experiments

**Scheme 2:**
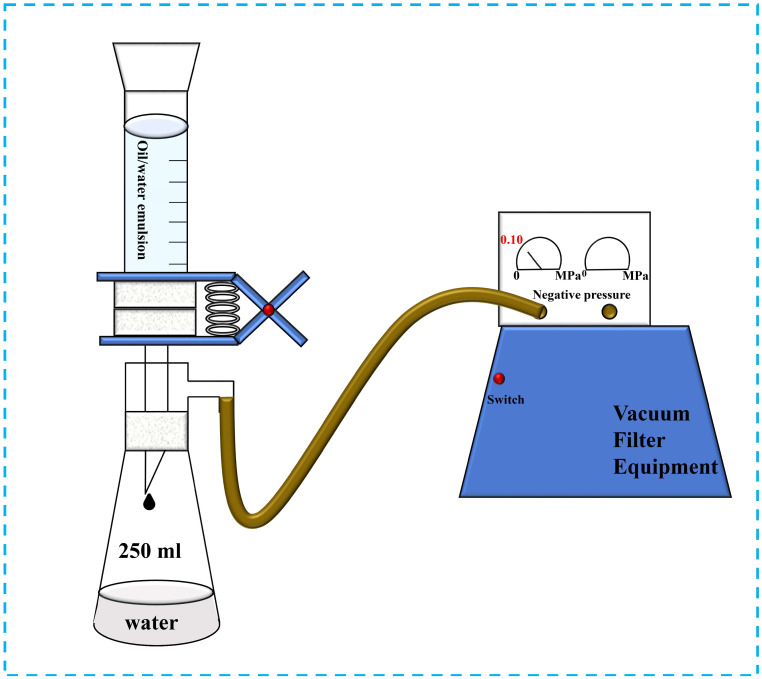
Schematic diagram of the emulsion separation experiments.

The as-prepared membrane was fixed in a vacuum filter apparatus (Scheme 2). The emulsion was poured directly onto the prepared fiber membrane, and the water was immediately permeated under negative pressure of 0.10 MPa. The water permeation flux *J* (L/m^2^ h) was calculated from Equation (1):

(1) $$J=\frac{V}{A\times \Delta t}$$

where *V* is the permeate volume of the water (L), *A* is the effective membrane area (m^2^), and Δ*t* is the permeate time (h). Oil rejection was determined by a UV spectrophotometer (UH4150, HITACHI).

### 2.6. Instruments and Characterization

The membrane surface information of chemical composition and bonding environment was measured by Fourier Transform infrared spectroscopy (FT-IR, BRUKER, Bremen, Germany) and X-ray photoelectron spectroscopy (XPS, Thermo Fisher K-alpha, Waltham, MA, USA). The morphologies of the prepared fiber membranes and ZIF-8 were characterized by a field-emission scanning electron microscope (FESEM, S-4800, Hitachi, Tokyo, Japan). The 3D surface structures of membranes were characterized by a true color confocal microscope (TCCM, CSM 700, Zeiss, Jena, Germany). The water contact angles of fiber membrane surfaces were measured by a contact angle meter (DSA-100, Kruss, Hamburg, Germany). An average value of water contact angle was obtained by measuring three different positions of each sample. The pore size of the fiber membrane was determined by capillary flow porometer (POROLUX 1000, Germany) and the porosity was obtained via the gravimetric method [47]. The mechanical property of hybrid membranes was measured at 25 °C by electron tensile instrument (YG061 F, Laizhou, China), and the tensile rate was 5 mm/min. Three measurements were performed for each sample. X-ray diffraction measurements (XRD, Bruker AXS D8, Germany) in the 2θ range from 10° to 80° were used to determine the crystalline structure of the membranes. The thermophysical properties of the fiber membranes were evaluated based on the thermogravimetric analysis equipment from 30 to 800 °C with a heating rate of 10 °C/min (TG, 209 F3 Tarsus, Netzsch Co., Selb, Germany).

## 3. Results and Discussion

### 3.1. Basic Properties of PPS Matrix

**Figure 2:**
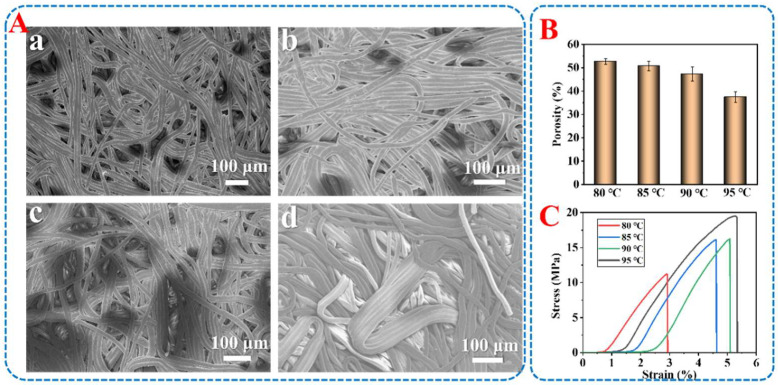
Basic properties of PPS matrix. (**A**) Surface SEM images of PPS fiber membranes (a: 80 °C; b: 88 °C; c: 90 °C; d: 95 °C); (**B**) porosity of PPS fiber membranes; (**C**) stress–strain curve of PPS fiber membranes.

In order to explore the best process condition and screen out the best PPS matrix to better support ZIF-8 and prepare better ZIF-8/PPS fiber membranes, as shown in Figure 2, the surface morphology, porosity determination, and mechanical properties of the nitrated PPS fiber membranes were tested. To observe the morphology evolution related to different hot-pressing temperatures (80 °C, 85 °C, 90 °C, 95 °C), the surface SEM images of PPS fiber membranes are shown in Figure 2A. By comparing the surface SEM images of the PPS fiber membrane obtained via hot pressing at different temperatures, it could be clearly seen from Figure 2A that the apparent pore size of the PPS fiber membrane was larger when the hot-pressing temperature was 80 °C, and the SEM image showed that the pore size could reach the micron level. However, the micron-level pore size was not conducive to the separation of small-molecule contaminants. With the increase in the hot-pressing temperature, the contact points and contact area between the fibers inside the PPS fiber membranes increased obviously, and even adhesion occurred between the fibers. The adhesion phenomenon caused the surface pore size of the fiber membranes to gradually decrease, and the reduction in the pore size was conducive to the improvement of the separation ability of the fiber membranes. At the same time, the thermal cross-linking between the fibers was beneficial in increasing the tensile strength of the PPS fiber membranes. From the perspective of the size of the PPS fiber, the hot pressing made the surface fiber inside the PPS fiber membrane flat, which was because the molecular structure of the PPS fiber was stimulated by the dual stimulation of heat and force to cause the directional movement of the molecular chain.

The surface SEM images of the PPS fiber membranes indicated that the pore size of the PPS fiber membranes became smaller as the temperature of hot pressing increased. As we all know, porous materials have a wide range of applications in the field of separation, and flux and rejection are two important parameters for evaluating their separation performance [48,49,50,51]. As shown in Figure 2B, as the hot-pressing temperature increased, the porosity of the PPS fiber membranes decreased. This was because hot pressing caused thermal cross-linking between the fibers. The fiber connection was closed, and the gap between the fibers was reduced.

As shown in Figure 2C, because the mechanical properties of the separation membrane greatly affect its practical application performance, the mechanical properties of the PPS fiber membranes were studied. The stress–strain curve of the fiber membranes has two parts. One part is the nonlinear elastic region, and the reason for the nonlinear elastic region is the tensile orientation of the fiber membranes. The other part is the linear elastic region, where the fiber breaks until it fails completely. It can be seen from Figure 2C that when the hot-pressing temperature was 80 °C, the stress of the PPS fiber membranes was significantly smaller. This was because 80 °C did not reach the glass transition temperature of the PPS, and thermal cross-linking and setting did not occur between the fibers in the fiber membranes. As the hot-pressing temperature increased, the degree of cross-linking between fibers became larger, causing the increase in tensile strength of the fiber membranes. In addition, the nitrification process before hot pressing destroyed the molecular chain structure of PPS; on the other hand, the crystallinity of the PPS fiber membranes was improved after hot pressing. Therefore, the stress–strain curve of the PPS fiber membranes after nitrification showed brittle fracture characteristics. In view of the dual consideration of porosity and mechanical properties, we chose 90 °C as an optimal hot-pressing temperature based on a large number of experiments.

### 3.2. Functional Group Analysis

**Figure 3:**
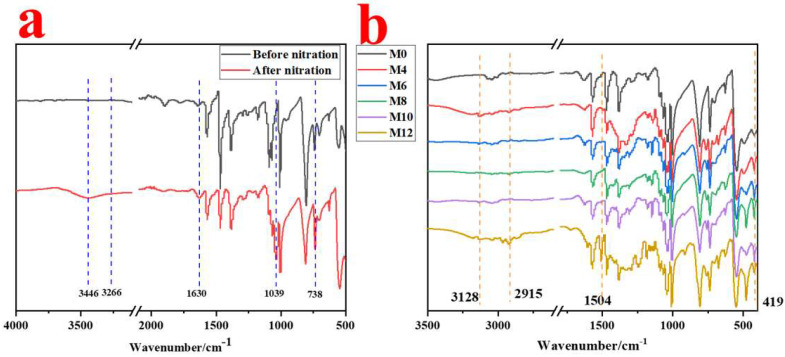
(**a**) FT-IR spectra of PPS non-woven fabrics before and after treatment with nitric acid solution; (**b**) FT-IR spectra of ZIF-8/PPS fiber membranes obtained after different ZIF-8 in situ growth times (M0: untreated; M4: 4 h; M8: 8 h; M10: 10 h; M12: 12 h).

As shown in Figure 3a, the peaks of PPS non-woven fabrics before treatment with the nitric acid solution were at 1630 cm^−1^ and 738 cm^−1^, which could be attributed to the stretching vibration peaks of the benzene ring in PPS. We found that the 1630 cm^−1^ and 738 cm^−1^ peaks of PPS non-woven fabrics after treatment with nitric acid solution became more intensive, and they corresponded to the bending vibration of N-H bonds of primary amine. Compared with pure PPS, two new peaks at 3446 cm^−1^ and 3266 cm^−1^ were attributed to primary amines. This was because the -NO_2_- group introduced undergoes a reduction reaction with -S- in PPS to reduce the -NO_2_- group into a -NH_2_- group. The peak at 1039 cm^−1^ was attributed to the stretching vibrations of -SO-, indicating the -S- had been oxidized via nitric acid. As shown in Figure 3b, the absorption peaks at 3128 cm^−1^ and 2915 cm^−1^ belonged to the stretching vibration peaks of the C-H bond in the methyl group and the imidazole ring, respectively. The C=N stretching vibration peak of the imidazole ring appeared at 1504 cm^−1^, and the stretching vibration peak of the Zn-N bond appeared at 419 cm^−1^. The peak from 1500 cm^−1^ to 1350 cm^−1^ belongs to the stretching vibration peak of the C=N bond on the imidazole ring. At the same time, the peak from 1350 cm^−1^ to 700 cm^−1^ belongs to the stretching vibration peak of the C-N bond on the imidazole ring. In summary, the FT-IR spectra of prepared composite fiber membranes confirmed the presence of ZIF-8 on the surface of the fiber membranes. In other words, we successfully prepared the ZIF-8/PPS fiber membranes.

### 3.3. Element Distribution Characterization

**Figure 4:**
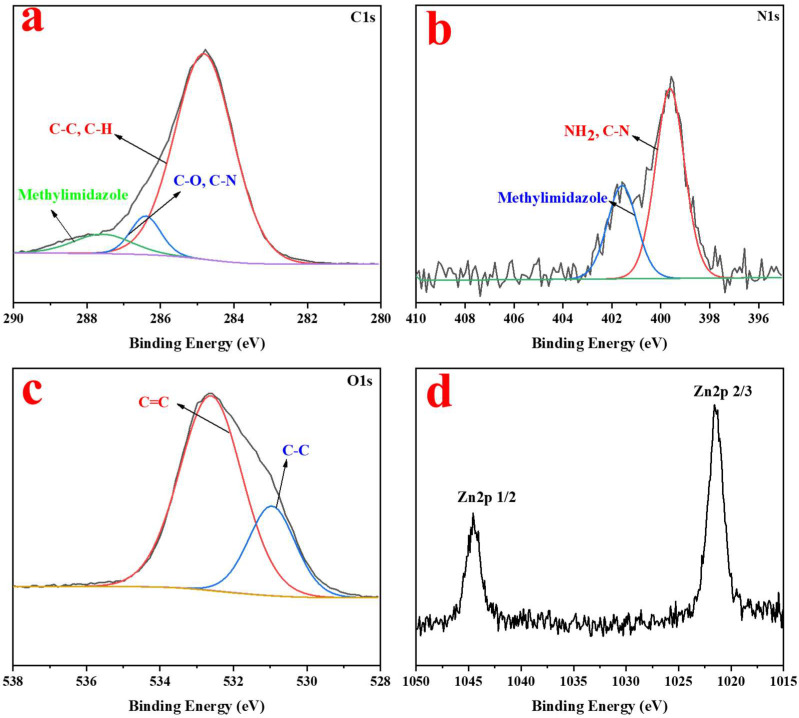
XPS spectra of the surface of the ZIF-8/PPS fiber membranes ((**a**): C1s; (**b**): N1s; (**c**): O1s; (**d**): Zn2p).

XPS was used to characterize the chemical composition of the surface of the ZIF-8/PPS fiber membranes. The XPS spectra of the surface of the ZIF-8/PPS fiber membranes are shown in Figure 4a–d, respectively. According to the XPS spectra of the surface of the ZIF-8/PPS fiber membranes, there were a number of bonds involving C, O, and N on the surface of the fiber membranes. The C1s spectrum was scanned in the binding energy range of 290 eV to 280 eV. It can be seen from Figure 4a that there were obvious characteristic peaks at 287.64 eV, 286.41 eV, and 284.82 eV. The peak at 287.64 eV corresponds to methylimidazole carbon, the peak at 286.41 eV corresponds to C-O and C-N, and the peak at 284.82 eV corresponds to C-C and C-H. It can be seen from Figure 4b that there are obvious characteristic peaks at 401.57 eV and 399.62 eV. The peak at 401.57 eV corresponds to methylimidazole nitrogen, and the peak at 399.62 eV corresponds to NH_2_ and C-N. It can be seen from Figure 4c that there are obvious characteristic peaks at 532.62 eV and 530.98 eV, which are related to C=O and C-O, respectively. In addition, according to the spectrum of Zn2p, it can be seen that there are obvious peaks at 1044.57 eV and 1021.57 eV. In summary, the ZIF-8 was successfully deposited on the surface of the PPS fiber membranes.

### 3.4. Membrane Morphology

**Figure 5:**
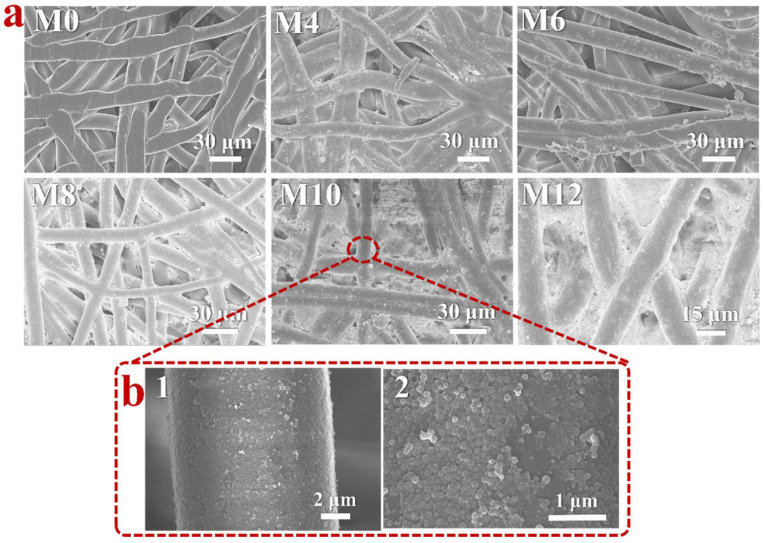
(**a**) SEM images of ZIF-8/PPS fiber membranes (M0: untreated; M4: 4 h; M8: 8 h; M10: 10 h; M12: 12 h); (**b**) SEM images of ZIF-8 attached to PPS fiber membranes (1: low magnification; 2: high magnification).

The SEM images of ZIF-8/PPS fiber membranes are shown in Figure 5a. It can be seen from Figure 2A and Figure 5a that the surface of the PPS fiber membranes before hydrophilic modification with nitric acid is relatively smooth. It is worth mentioning that the in situ growth strategy requires both stable fibers as the template and numerous active groups (hydroxyl and carboxyl) to anchor the MOFs [52]. In addition, the previous work of our research group can confirm that the nitrated PPS surface had a large number of functional groups, which makes it easy for ZIF-8 nanoparticles to grow in situ on the surface of the fiber membranes. As shown in Figure 5a, there are obvious differences in the surface microstructure of ZIF-8/PPS fiber membranes prepared under different in situ growth times. It can be seen from M4-M12 that there is a deposition layer of ZIF-8 on the surface of the hybrid fiber membranes. When the growth time was 4–6 h, the ZIF-8 crystals grown in situ were rare and the morphological structure was not obvious. When the growth time of ZIF-8 on membranes was 8 h, regular crystal particles could be seen on the surface of the PPS fiber membranes. As the ZIF-8 in situ growth time increased, the number of ZIF-8 nanocrystals increased. When the growth time of ZIF-8 on the PPS fiber membranes was 12 h, a dense ZIF-8 crystalline layer was formed, and the agglomeration of inorganic nanoparticles occurred. This was because the in situ growth system was in a static state, the growth of ZIF-8 crystals was completely free, and the accumulation of ZIF-8 particles was also free. Therefore, ZIF-8 particles will have a large amount of aggregation under the condition of no external force disturbance. The MOFs were filled between the PPS fibers, and the ZIF-8 structure itself has nano-sized pores, thus forming a micro/nano structure, and it can be seen from Figure 5a that the micro/nano structure was stacked on the surface of the PPS fiber membranes with the introduction of ZIF-8. The existence of a micro/nano structure made the pores on the PPS fiber membranes more tortuous. The nanostructure of ZIF-8 and the microporous structure of the PPS fiber membranes formed a synergistic effect. The interesting synergistic effect can not only diversify the pore size of the hybrid membranes but also improved the separation performance of the PPS fiber membranes. The SEM images of ZIF-8 attached to PPS fiber membranes are shown in Figure 5b. As shown in Figure 5b, ZIF-8 nanoparticles were regular hexagons, and they were closely and regularly arranged on the surface of the PPS fiber membranes.

### 3.5. Crystallinity Analysis

**Figure 6:**
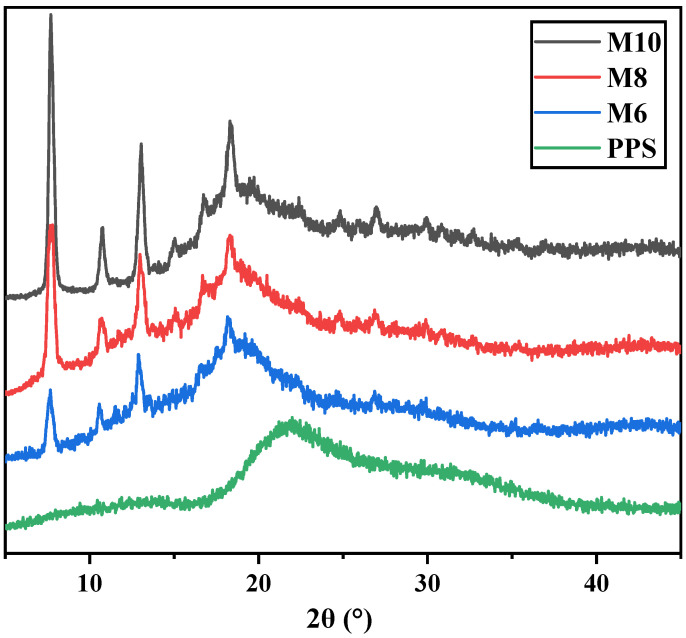
XRD patterns of PPS and ZIF-8/PPS fiber membranes.

Figure 6 shows the XRD patterns of PPS and ZIF-8/PPS fiber membranes. The changes in polymer crystallization of the PPS fiber membrane and ZIF-8/PPS composite fiber membrane before and after the reaction were studied. The PPS fiber membranes had C crystal peaks at 2θ = 13° and 22°, and the peaks were more obvious. This was because the originally unstable thermodynamic state in the PPS fiber disappeared when the PPS non-woven fabrics were made into PPS fiber membranes via hot pressing. On the other hand, when the hot-pressing temperature was above the glass transition temperature of PPS, the nascent PPS fibers gradually began to crystallize, forming a certain crystalline structure in the PPS fibers. This was also the reason why PPS fibers after hot pressing can maintain good dimensional stability at higher temperatures. The ZIF-8/PPS fiber membranes had obvious characteristic peaks at 8.0°, 10.3°, 12.6°, and 17.9°. Moreover, the characteristic peak intensities of the ZIF-8/PPS composite fiber membranes at 2θ = 18° and 27° were higher than that of the PPS fiber membrane, which may be the result of the superposition of the peak intensities of PPS and ZIF-8. As the growth time of ZIF-8 increased, the peak intensity gradually increased. The XRD analysis results indicated that ZIF-8 had grown on the surface of the PPS fiber membranes, and this result also illustrated the successful preparation of ZIF-8/PPS fiber membranes.

### 3.6. Porosity and Roughness Measurements

**Figure 7:**
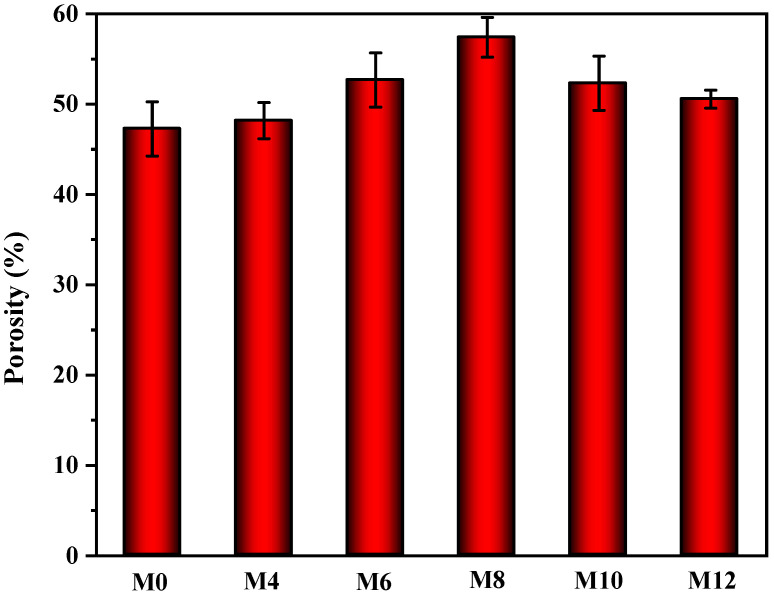
The porosity of ZIF-8/PPS fiber membranes (M0: untreated; M4: 4 h; M8: 8 h; M10: 10 h; M12: 12 h).

Figure 7 shows the porosity of the ZIF-8/PPS composite fiber membranes. It can be seen from Figure 7 that the porosity of the ZIF-8/PPS composite fiber membranes first increases and then decreases with the increase in ZIF-8 in situ growth time. When ZIF-8 growth time was less than 8 h, the porosity of the ZIF-8/PPS composite fiber membranes increases for two reasons: on the one hand, the micro/nano structure composed of ZIF-8 nanocrystals increased the porosity of the fiber membranes; on the other hand, when the ZIF-8 growth time was not long enough, the liquid retention performance of the ZIF-8/PPS composite fiber membranes was improved with the increase in ZIF-8 growth time. When the ZIF-8 in situ growth time was long enough (more than 8 h), the number of ZIF-8 increased, forming a dense crystalline layer of ZIF-8, and agglomeration occurred. The agglomeration blocked the pores of the fiber membrane. In other words, the addition of a large amount of ZIF-8 occupies the pore space of the membrane, thus reducing the porosity.

**Figure 8:**
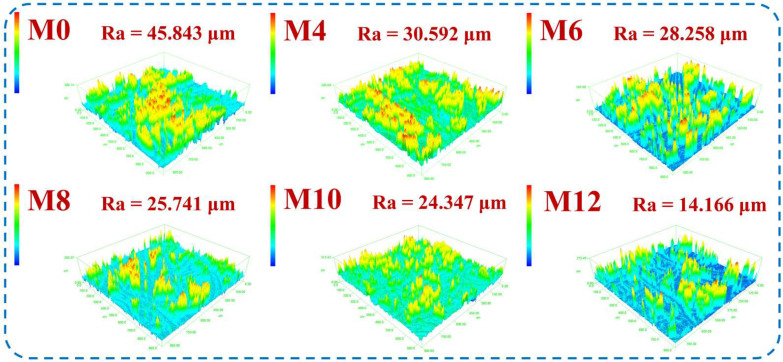
The 3D surface roughness of ZIF-8/PPS fiber membranes (M0: untreated; M4: 4 h; M8: 8 h; M10: 10 h; M12: 12 h).

Compared with PPS fiber membranes, the roughness of the composite membranes decreased significantly. It can be observed from Figure 8 that the roughness of the PPS fiber membrane was 45.843 μm. As the growth time of ZIF-8 increased, the surface roughness of the fiber membrane was significantly reduced. This was because the internal structure of the PPS fiber membranes was a kind of complex interlaced and irregularly arranged messy network structure, and the fibers undergo thermal cross-linking behavior after hot pressing. The heat-cross-linked fibers form depressions, so the roughness of the PPS fiber membrane is relatively large. The introduction of ZIF-8 can fill the depressions in the PPS fiber membrane, and the roughness of the fiber membranes begins to decrease. It was found that as the growth time of ZIF-8 increases, the continuity of the ZIF-8 layer on the surface of the fiber membranes becomes better and better, causing the reduction in the roughness. The membrane surface roughness results indicated that the amount of ZIF-8 increases as ZIF-8 in situ growth time increases. Since ZIF-8 is a polygonal nano-scale crystal, its particles are irregularly stacked on the surface of the PPS fiber membrane to form a micro/nano structure. The introduction of ZIF-8 reduced the roughness of the PPS fiber membranes and improved the hydrophilicity of the fiber membranes. More importantly, this interesting membrane surface modification strategy also improved the oil/water separation efficiency of PPS fiber membranes.

### 3.7. Thermogravimetric Analysis

**Figure 9:**
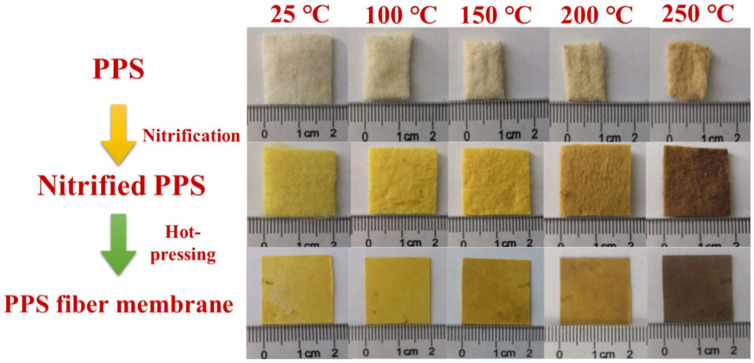
High-temperature resistance experiment diagram of PPS matrix.

In order to test the high-temperature resistance of the PPS, as shown in Figure 9, the thermal shrinkage rate of PPS non-woven fabric samples at different temperatures was tested (100 °C, 150 °C, 200 °C, 250 °C), and it was found to be 40%, 45%, 45%, and 50%, respectively, showing a continuous upward trend. At the same time, the PPS non-woven fabrics showed obvious curl with the increase in the test temperature, and the color gradually changed from white to yellow. On the one hand, the above thermal shrinkage phenomenon is due to the high-speed hot air used to draw the PPS fiber during the molding process of the PPS non-woven fabric, which led to large internal stress in the PPS fiber. When the ambient temperature is higher than the glass transition temperature of PPS, the PPS molecular chain relaxes so that the PPS fiber membrane undergoes macroscopic shrinkage after being heated. On the other hand, the crystallinity of PPS non-woven fabric prepared via the melt-blown is low. PPS in a high-temperature environment will crystallize, and the higher the temperature, the faster the crystallization rate of PPS and the tighter molecular chain arrangement. The crystallization behavior increased the thermal shrinkage rate of PPS fibers.

The nitrated PPS non-woven fabrics had almost no change in size when the temperature was lower than 200 °C. When the temperature reached 250 °C, the heat shrinkage rate of the nitrated PPS non-woven fabrics was only 10%. More importantly, the PPS fiber membranes obtained via hot pressing did not show any change in membrane size after being treated at 250 °C for 6 h. The reason was that the heat setting process can promote the fibers in the fiber membrane to tend to a thermodynamic equilibrium state, ensuring that the fiber membrane formed by the fiber stack can maintain good dimensional stability at a higher temperature. In this study, the PPS fiber was hot pressed under the condition of being higher than its glass transition temperature. The post-processing can promote the movement of frozen PPS molecular chains, so that the PPS fiber membranes can reach a thermally stable state.

**Figure 10:**
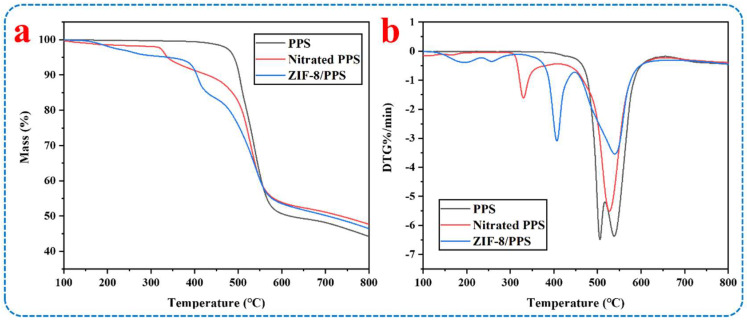
(**a**) TG curves of PPS non-woven fabrics, PPS non-woven fabrics after nitrification, and ZIF-8/PPS fiber membranes; (**b**) DTG curves of PPS non-woven fabrics, PPS non-woven fabrics after nitrification, and ZIF-8/PPS fiber membranes.

The TG was used to test the high-temperature stability of PPS non-woven fabrics, PPS non-woven fabrics after nitrification, and ZIF-8/PPS fiber membranes. The results are shown in Figure 10. PPS non-woven fabrics have no obvious quality loss below 500 °C, showing good thermal stability. Afterward, an obvious weight loss peak appears at 500 °C to 600 °C. A small weight loss peak appears for nitrated PPS at 300 °C, and the part of the quality loss was mainly caused by the evaporation of H_2_O and the reduction of a very small amount of HNO_3_ attached to the surface of the nitrified PPS fiber membrane. The weight loss is most obvious between 500 °C and 600 °C, and this part of the weight loss was mainly caused by the decomposition of nitrated PPS. It is worthy of our attention that the weight loss of the prepared composite membrane is most obvious between 500 °C to 800 °C. At 500 °C to 800 °C, the ZIF-8 decomposes and the structure of the ZIF-8/PPS fiber membranes collapses. From the above TG results, it can be seen that the ZIF-8 and PPS matrix have no significant weight loss below 200 °C, and the more obvious decomposition phenomenon occurred when the temperature was higher than 500 °C. On one hand, the TG data illustrate the successful construction of the composite membrane structure, that is, the successful preparation of the ZIF-8/PPS fiber membranes. On the other hand, they illustrate that the ZIF-8/PPS fiber membranes can be used in the field of high-temperature separation.

### 3.8. Wettability of Membrane Surface

**Figure 11:**
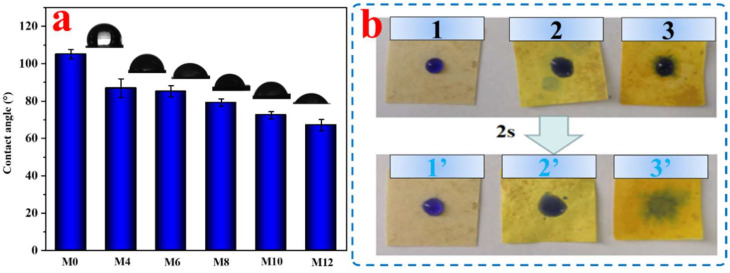
Wettability of ZIF-8/PPS fiber membranes. (**a**) Water contact angle of ZIF-8/PPS fiber membranes (M0: untreated; M4: 4 h; M8: 8 h; M10: 10 h; M12: 12 h); (**b**) the changes in water droplets after standing for 2 s on different surfaces (1 and 1′: untreated PPS; 2 and 2′: nitrated PPS; 3 and 3′: ZIF-8/PPS).

Figure 11 shows the test results of the wettability of the ZIF-8/PPS fiber membranes. To explore the influence of different ZIF-8 in situ growth time on the wettability of ZIF-8/PPS fiber membranes, we measured the water contact angle of the ZIF-8/PPS fiber membranes. It can be seen from Figure 11a that the contact angle of the untreated PPS fiber membrane is 105.1°, which is hydrophobic. After modification for different reaction times, the contact angle of the fiber membrane showed a downward trend. This is because the stack of many ZIF-8 nanoparticles can fill the gully structure of the PPS fiber membranes, and the reduction in the gully structure was beneficial in improving the wettability of the fiber membranes. Therefore, as ZIF-8 in situ growth time increased, the hydrophilicity of the ZIF-8/PPS fiber membranes gradually increased. When the ZIF-8 in situ growth time was 12 h, the contact angle of the composite membranes reached 62.4°. Water droplets were dropped on the untreated PPS fiber membrane, the nitrated PPS fiber, and the ZIF-8/PPS fiber membrane, respectively. After keeping for 2 s, the shape of the water droplet on the untreated fiber did not change, and the contact angle of the water droplet on the nitrated fiber became smaller. Furthermore, the water droplets on the ZIF-8/PPS fiber membranes were completely absorbed. The phenomenon showed that the introduction of ZIF-8 greatly improves the hydrophilicity of the PPS fiber membranes.

### 3.9. Water Flux and Oil/Water Separation Performance

The oil/water separation performance of the organic–inorganic hybrid PPS fiber membrane has been reported by other researchers [53]. The excellent wettability and unique membrane structure made ZIF-8/PPS fiber membranes have broad application prospects in the field of oil/water separation. This proves that the large-scale application of PPS fiber membrane materials in the field of oil/water separation is feasible. Therefore, we prepared an oil-in-water emulsion of chlorobenzene to test the separation performance of the emulsion. Before the emulsion separation experiment, the ZIF-8/PPS fiber membranes were pre-wetted with deionized water. Due to the hydrophilic nature of the ZIF-8/PPS fiber membranes, the water molecules in the oil-in-water emulsion quickly penetrated into the fiber membrane under the drive of pressure, gravity, and capillary force, forming a water layer on the surface of the ZIF-8/PPS fiber membranes. The coupling of the water layer and the fiber structure prevented direct contact between the chlorobenzene molecules and the fiber membrane, thereby realizing the separation of the emulsion.

**Figure 12:**
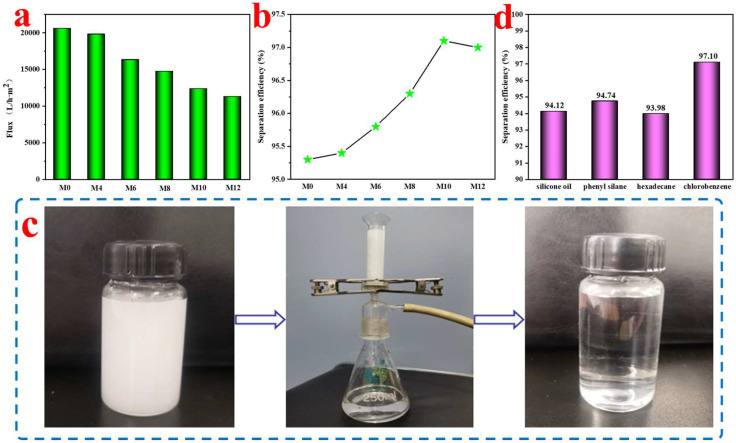
Water flux and oil/water separation performance of ZIF-8/PPS fiber membranes. (**a**) Water flux test of ZIF-8/PPS fiber membranes (M0: untreated; M4: 4 h; M8: 8 h; M10: 10 h; M12: 12 h); (**b**) oil/water separation performance (M0: untreated; M4: 4 h; M8: 8 h; M10: 10 h; M12: 12 h); (**c**) digital photos of M10 in the separation process of chlorobenzene; (**d**) separation performance of ZIF-8/PPS fibers for different emulsions.

It can be seen from Figure 12a,b that as the in situ growth time of ZIF-8 continues to increase, the flux of the ZIF-8/PPS fiber membranes decreased and the separation efficiency increased, which was also in line with the “Trade-off” effect. When ZIF-8 in situ growth time was 10 h, the separation efficiency of ZIF-8/PPS fiber membranes was higher (Figure 12c shows digital photos of M10 in the separation process of chlorobenzene). At the same time, considering the selectivity and permeability of the membrane, the distribution of ZIF-8, and the water wettability of ZIF-8/PPS fiber membranes, M10 was selected as the best choice to be used for oil/water separation. We further explored the oil/water separation performance of M10 in other different emulsions. Figure 12d shows the separation efficiency of M10 for four different types of oil/water emulsions (silicone oil, phenyl silane, hexadecane, and chlorobenzene). It can be seen from Figure 12d that all separation efficiencies were above 90%. It is worth noting that the separation efficiency of the ZIF-8/PPS fiber membranes for chlorobenzene emulsion is as high as 97.01%. This was mainly because the ZIF-8/PPS fiber membranes had good hydrophilicity and uniform pore size distribution, and the particle size of the chlorobenzene/water emulsion was larger than that of the other three emulsions.

### 3.10. Corrosion Resistance Performance and Recycling Performance

**Figure 13:**
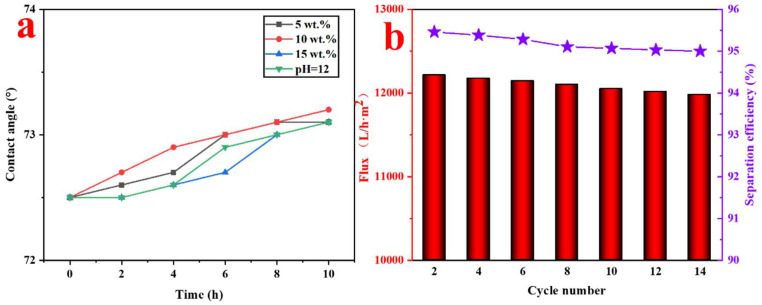
(**a**) PPS/ZIF-8 fiber membranes’ resistance to high alkali and salt solutions; (**b**) recycling performance of PPS/ZIF-8 fiber membranes.

In practice, the conditions that the fiber membranes are exposed to are not an ideal neutral environment. In order to test whether the hydrophilic properties of the ZIF-8/PPS fiber membranes under extreme environments will be affected by external stimuli, the hydrophilic ZIF-8/PPS fiber membranes were placed in a high alkali solution (pH = 12) and salt solutions of different concentrations (5 wt.%, 10 wt.%, and 15 wt.% NaCl solutions). As shown in Figure 13a, the contact angle of the ZIF-8/PPS fiber membranes did not change much and remained at about 73° after being immersed in high alkali and salt solutions for 10 h. The result indicated that the hydrophilicity of the ZIF-8/PPS fiber membranes was very stable.

The cyclic stability of the membranes characterizes the long-term serviceability of the membranes. Figure 13b shows the cyclic separation performance of fiber membranes. After each oil/water separation test was completed, the fiber membranes were cleaned with ethanol and deionized water, and then the next cycle was entered. The separation efficiency and flux of the fiber membranes were tested every two times, and 14 cycles of testing were performed. As shown in Figure 13b, the permeation flux was basically stable at 12,000 L/m^2^·h with the increase in the number of cycles. At the same time, the separation efficiency of the ZIF-8/PPS fiber membranes was always maintained above 95% (purple stars), which showed that the ZIF-8/PPS fiber membranes had good cycle stability.

## 4. Conclusions

In summary, the nitrated PPS fiber membranes were prepared via nitrification reaction and hot-pressing post-treatment, using PPS non-woven fabric as the base material. The ZIF-8/PPS fiber membranes were obtained by inserting ZIF-8 nanoparticles on the surface of the PPS fiber membrane via in situ growth. The distribution of ZIF-8 nanoparticles in the PPS fiber membranes was adjusted by changing the ZIF-8 in situ growth time to control the oil/water separation effect of the ZIF-8/PPS fiber membranes. The results show that when the ZIF-8 in situ growth time was 10 h, the contact angle, the porosity, and the pure water flux of the ZIF-8/PPS fiber membranes were 72.5°, 52.3%, and 12,351 L/h·m^2^, respectively. More importantly, the separation efficiency of M10 was 97%, and the oil/water separation efficiency reached 95% after 14 cycles. The composite membranes prepared in this study provide a fast and effective strategy for oil/water separation in extreme environments (high salt or alkaline conditions).
